# Fluid Resuscitation in Patients With Traumatic Brain Injury: A Comprehensive Review

**DOI:** 10.7759/cureus.43680

**Published:** 2023-08-18

**Authors:** Mayuri G Sontakke, Nikhil G Sontakke, Akhilesh S Parihar

**Affiliations:** 1 Accident Trauma Care and Technology, Jawaharlal Nehru Medical College, Datta Meghe Institute of Higher Education and Research, Wardha, IND; 2 Health Sciences, Jawaharlal Nehru Medical College, Datta Meghe Institute of Higher Education and Research, Wardha, IND; 3 Emergency Department, Jawaharlal Nehru Medical College, Datta Meghe Institute of Higher Education and Research, Wardha, IND

**Keywords:** brain trauma injury, brain trauma, hemorrhagic shock, traumatic brain injury, fluid resuscitation

## Abstract

Patients with traumatic brain injury (TBI) or head trauma present challenges for emergency physicians and neurosurgeons. Traumatic brain injury is currently a community health issue. For the best possible care, it is crucial to understand the various helpful therapy techniques in the pre-operative and pre-hospital phases. The initial rapid infusion of large volumes of mannitol and a hypertonic crystalloid solution to restore blood pressure and blood volume is the current standard of care for people with combined hemorrhagic shock (HS) and traumatic brain injury. The selection and administration of fluids to trauma and traumatic brain injury patients may be especially helpful in preventing subsequent ischemic brain damage because of the hemodynamic stabilizing effects of these fluids in hypovolemic shock. Traumatic brain injury is an essential factor that may lead to disability and death in a patient. Traumatic brain damage can develop either as a direct result of the trauma or as a result of the initial harm. Significant neurologic problems, such as cranial nerve damage, dementia, seizures, and Alzheimer's disease, can develop after a traumatic brain injury. The comorbidity of the victims may also be significantly increased by additional psychiatric problems such as psychological diseases and other behavioral and cognitive sequels. We review the history of modern fluid therapy, complications after traumatic brain injury, and the use of fluid treatment for decompressive craniectomy and traumatic brain injury.

## Introduction and background

The term "traumatic brain injury" (TBI) is most often used to describe brain dysfunction brought on by external trauma. Many types of traumatic injuries happen worldwide, and traumatic brain injury is one of the most contentious health issues in modern society [1]. The Centers for Disease Control report that between 2001 and 2010, there was an increase in hospitalizations and overall average rates of traumatic brain injury-related visits to emergency departments [2]. Individually, nevertheless, fewer people have died as a result of traumatic brain injuries over the same time. This drop is probably partly attributable to greater awareness, the formalization of care and guidelines, and significant technological advances in treatments today. Therefore, it is likely that the reported rates of TBIs are lower overall. We should also be aware that some traumatic brain injuries never receive medical attention [3]. The age groups with the highest rates of traumatic brain injury are often those between zero-four years and 15-24 years. Another rise in incidence is seen in people over 65 years old. Falls and car accidents are the two main factors that cause traumatic brain injuries globally [4]. Head injuries are common in emergency departments worldwide, with over two million visits in North America and 400,000 in Europe [5]. A serious public health issue and the primary cause of mortality and disability is traumatic brain injury. It often occurs with hemorrhagic shock [6]. The lack of cerebral auto-regulation, which led to further ischemia injury to the already fragile brain, as well as the adverse effects of traumatic brain injury on the typical compensatory response to hemorrhagic shock, may both play a role in the mechanism for unfavorable outcomes in people with combined hemorrhagic shock and traumatic brain injury [7]. Since there is no way to reverse the primary injury, therapeutic techniques must instead concentrate on preventing secondary harm by avoiding hypoxia and hypotension and maintaining healthy cerebral perfusion pressure (CPP), which serves as a stand-in for healthy cerebral blood flow (CBF) [8]. Massive doses of crystalloids are currently infused quickly to restore blood pressure and blood volume in patients with combined traumatic brain injury and hemorrhagic shock [9,10].

An essential part of surgical treatment is administering perioperative fluids, although this process is frequently not completely understood and is still primarily empirical. There are lingering concerns regarding its effectiveness and potential consequences [6,11]. Fluid therapy (FT) is a treatment that uses fluids [12]. Optimizing the circulatory system is the ultimate goal of fluid management to ensure that organs receive enough oxygen [13]. Colloid and crystalloid solutions are the two main intravenous fluid classifications. They have significantly dissimilar physiological, chemical, and physical traits [6]. Colloid solutions are made up of natural substances like albumin or artificial ones like dextran, hydroxyethyl starches, and gelatins. Goal-directed fluid therapy, which has been proven effective and aims to maximize oxygen supply and cardiac output, improves the outcome of patients undergoing risky surgeries [14]. Most of the information provided here is drawn from articles on the fluid management of trauma patients. Primary research is on pre-operative fluid treatment for destructive craniectomy. It is debatable how fluids should be managed for patients having elective major surgery, such as neurotrauma surgery [15-17]. Numerous pathophysiological alterations that affect fluid homeostasis's regular effectiveness occur during the perioperative period. Despite this, the perioperative fluid prescription is frequently subpar because it is founded on a need to understand the distribution and requirements for water and electrolytes [6,18-21]. Blood components, crystalloids, and colloids are necessary during surgery to replenish continuing losses and preserve circulatory stability to ensure adequate tissue perfusion [22,23]. The perioperative fluid requirements are influenced by the seriousness of the situation, the scope and length of the procedure, concomitant situations, and the host's response to injury [6]. In addition to replacing fluid loss or maintaining fluid balance, intravenous fluids may be explicitly used to correct an existing electrolyte or acid-base disorder [24]. The aim of this work is to review the current topics of fluid management in patients with traumatic brain injury and the fluid treatment for decompressive craniectomy.

## Review

Methodology

**Figure 1:**
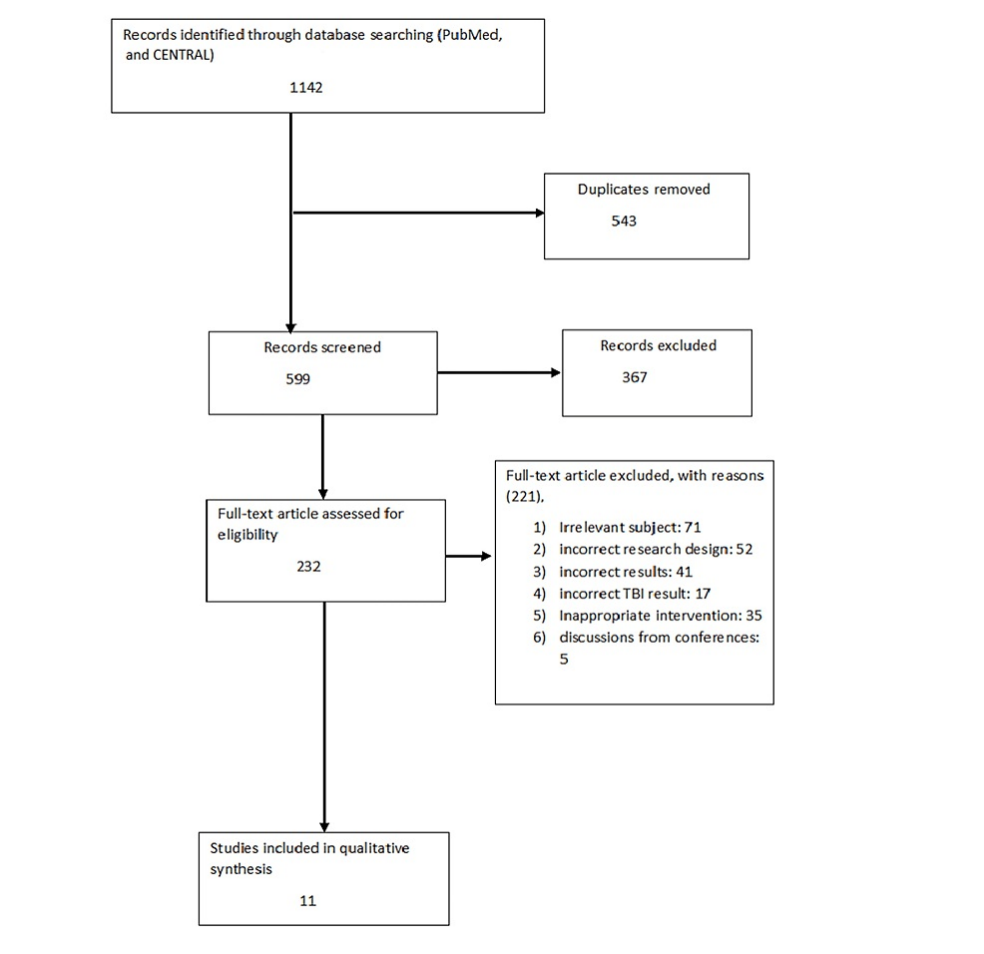
Prisma flow diagram of the search strategy

We undertook a search through PubMed, CENTRAL and other government sources in November 2022 using keywords such as "traumatic brain injury", "fluid resuscitation", "brain injury", "brain trauma", and "colloid solutions"((( traumatic brain injury [Title/Abstract]) OR ("traumatic brain injury" [MeSH Terms]), ("fluid resuscitation" [Title/Abstract])) OR ("fluid resuscitation" [MeSH Terms]), (("brain injury" [Title/Abstract]) OR ("brain injury" [MeSH Terms]) AND ("brain trauma" [Title/Abstract]) OR ("brain trauma" [MeSH Terms]). One reviewer independently checked the papers retrieved based on title and abstract against the inclusion criteria before moving on to the full texts. Another reviewer also reviewed 20% of these studies to validate inclusion studies. The selection of studies (Figure 1) depended on the following inclusion criteria: (1) patient with brain injury; (2) patient who had fluid resuscitation; (3) patient with brain trauma (4) English language; (5) systematic reviews. The following were the exclusion criteria: (1) case study; (2) non-English language articles; (3) opinion articles; (4) technical reports; (5) surveys.

The evolution of modern fluid therapy

The importance of intravenous fluid therapy in treating cholera initially emerged in the 1830s, thanks to William Brooke O'Shaughnessy's findings on the blood observations of his suffering cholera patients [6,25]. It was determined that those individuals' blood was thick and dense and suffering from a water deficiency [26]. For surgical patients, a 0.9% physiologic saline solution replenished the lost corporal fluid. Intravenous fluid therapy was first used during surgery to offset the harmful effects of anesthesia in the 1880s. As a result, clinical benefits were noticed, although saline's adverse effects were also discernible [25,27]. Fluid treatment did not initially become widely accepted since the use of various water and salt mixtures, along with the unsanitary practices of the time, did not show safety [6]. Sidney Ringer invented Ringer's solution in 1880 after seeing the various protoplasmic activities of salts of sodium, calcium, potassium, and chloride [12]. Applying an animal model of hemorrhagic shock, George Crile investigated the remedy in 1899 and advised applying it warm. Primitive saline and colloid solutions were used in the First World War to treat combat injuries. Gum Arabic, a natural colloid made by wide converter cannons from the Acacia Senegal tree, was a high-point colloid [6]. Rudolph Matas invented the intravenous "drip" in 1924. To prevent the hyperchloremic acidosis brought on by using Ringer's solutions, Hartmann and Senna added sodium lactate in 1930. This made it possible to connect sodium to excess chloride. The Hartman solution, or Ringer lactate, was created because the lactate metabolism was facilitated. The work of Hartmann, Ringer, and others highlighted the significance of the intravenous fluid composition and laid the groundwork for the balanced solutions currently in use [28,29]. Blood and plasma were widely distributed during the Second World War, even on the battlefield, to extend the lives of wounded soldiers. Fluid therapy (FT) has generally undergone constant change based on current developments [12]. The best-known surgeons, Moore and Shires, vigorously discussed the origin of post-operative oliguria during the 1940s and 1950s as the metabolic response to injury was progressively researched [29]. Fluid restriction has been studied in recent fluid treatment studies. Goal-directed fluid treatment was made possible thanks to Shoemaker and colleagues' groundbreaking work on fluid administration to attain supranormal cardiorespiratory performance [6]. Intravenous fluid therapy administration is now governed by physiological principles with a fresh appreciation of the lessons gained from history and the development of balanced solutions due to the increased emphasis on perioperative fluid therapy [29]. Fluid treatment has received particular attention as military medicine has advanced, and it has grown in significance when paired with chemotherapy; however, without any definite thoughts regarding the right volume, fluid treatment still sparks significant debates today [6].

Crystalloids

Small, water-soluble molecules that make up a crystalloid fluid can quickly diffuse across semi-permeable barriers. These solutions' tonicity (osmolality with plasma) and sodium content (which affects how they are distributed throughout the body compartments) together greatly influence their features [30]. They redistribute across the compartment of extracellular fluid, of which interstitial fluid makes up 75%. This implies that four liters of crystalloid are needed to compensate for a one-liter loss in blood [31]. Studies have revealed that patients with normovolaemic and hypovolaemic conditions have different volume kinetics of infused crystalloid solutions [32]. After 30 minutes of isotonic saline solution intravenous infusions, which only contribute up to a third of the volume used in regular patients to the increase in intravascular space, only 16% of the intravascular space is left. Due to redistribution into and quick clearance from the extracellular fluid, the amount of crystalloid needed to replenish acute blood loss stays three-to-four times higher [31].

Colloids

Colloids are liquids that impose oncotic pressure on semi-permeable membranes because they are composed of larger, more soluble molecules that are difficult to penetrate. Through osmosis, water is retrieved from the interstitial and international classifications of functioning based on their shape, molecular weight, capillary permeability, and ionic charge. They exit the intravascular region and act for a specific time [30]. All colloids except albumin are polymers and comprise particles of various molecular weights [6]. Because of their increased osmolality, possibly by more than the volume administered, they may increase plasma volume; therefore, the name "plasma expanders" [31]. According to studies, they can significantly inhibit the activity of clot formation [33,34].

Traumatic brain injury and fluid therapy

Oedema development is not significantly impacted by clinically appropriate fluid restriction. The first investigation on human fluid therapy showed that decreasing the standard maintenance volume by 50% for neurosurgical patients causes an increase in serum osmolality over about a week [35]. Therefore, the previous idea of the advantage of fluid blockage resulted from an osmotic gradient that rises with time [6]. According to the information that is now available, volume expansion and replacement will not affect cerebral oedema as long as normal serum osmolality is preserved and cerebral hydrostatic pressures are not noticeably raised as a result of elevated proper ventricular pressures and real volume overload. The osmolality of the chosen fluid is essential. However, it needs to be clarified if this is accomplished with crystalloid or colloid [36]. A penetrating or blunt injury causes the blood-brain barrier's typically tightly intact endothelium to be destroyed mechanically and automatically in traumatic brain injury [37]. This enables unchecked serum and fluid protein transport into the brain parenchyma, ultimately resulting in vasogenic cerebral oedema and elevated intracranial pressure. It has been demonstrated that severely ill patients have higher albumin leakage through the capillary wall [38]. This elevated albumin extravasation in the brain may worsen cerebral oedema and increase interstitial oncotic pressure [6].

Fluid treatment for decompressive craniectomy

Based on experience, it is suggested that decompressive craniectomy in traumatic epidural hematoma patients improves these patients' outcomes [39]. The general rules for fluid management within the context of enhanced recovery and recommendations for the enhanced recovery partnership are listed below: in pre-operative patients (provide carbohydrate drinks; avoid bowel preparation; maintain excellent pre-operative hydration); in perioperative patients (utilize fluid management technologies to administer personalized fluid therapy with a goal in mind; avoid using too much salt or water when making crystalloids; if maintenance fluid is used, it should not exceed 2 ml/kg/hr, without counting any medicine infusions; hyperchloraemic acidosis can be reduced by using an isotonic balanced electrolyte, such as Hartmann's solution) and in post-operative patients (avoid post-operative intravenous fluids whenever feasible, encourage early drinking and eating and ask yourself " why do we administer fluids? ", consider oral fluids before intravenous ones, and prescribe them, fluids for resuscitation and goal-directed fluid treatment) [40].

Physiological reactions in the perioperative stage

The effects of surgery alone and the resulting adjustments to the hormonal environment inside the body are exacerbated in critically ill patients by a systemic inflammatory response that leads to capillary leak development. As a result, the interstitium endures losses that are challenging to balance and frequently display oedema. Additionally, nutritional support and actions that alter acid-base homeostasis can actively or inadvertently influence the fluid and electrolyte imbalances that result in severe illness [41]. The effects of surgery include changes to fluid balance, an increase in cardiac output and oxygen delivery, as well as a systemic inflammatory response that increases oxygen consumption. Failure to recuperate from surgery in a way that meets metabolic demands is linked to higher morbidity and death [30]. Surgery and trauma stress responses entail a variety of physiological responses. A significant effect is the stimulation of the renin-angiotensin-aldosterone pathway, which causes altered fluid balance, increased sodium and fluid retention, and decreased urine output [5]. Additionally, the activated inflammatory response causes vasodilatation and enhanced capillary wall permeability. This shortens the time that fluids are administered intravenously and causes more fluid to leak from capillaries into the interstitial tissues. As a result, the body's ability to handle fluids during the perioperative period is significantly altered and should be carefully considered while prescribing fluids [30]. The limitations of the review include a lack of discussion on drugs used to treat TBI and other management. The keywords used couldn’t find the articles. Although many studies have examined the complications of TBI, the effects of TBI, and the pathophysiology of TBI, there is a lack of research on fluid resuscitation in patients with traumatic brain injury and their management.

Table 1 discusses the characteristics of the articles included in the review.

**Table 1:** Summary of the articles included in the review TBI: traumatic brain injury; HES: hydroxyethyl starch

Author	Year	Journal	Country	Outcomes
Ahmed S et al, [1]	2017	Indian Journal of Psychological Medicine	USA	Maximize neurocognitive recovery and functional independence from secondary traumatic brain injuries.
Rutland-Brown W et al, [4]	2006	Journal of Head Trauma Rehabilitation	USA	Traumatic brain injury (TBI) rates the highest in young children and men, with falls and motor vehicle traffic being the leading causes.
Neville KA et al, [28]	2010	The Journal of Pediatrics	Australia	Isotonic saline solution reduced hyponatremia risk without fluid restriction.
Srinivasa S et al, [29]	2012	Annals of Surgery	New Zealand	Goal-directed fluid therapy improves cardiorespiratory function by addressing supranormal indices.
Cook SC et al, [30]	2009	Anaesthesia & Intensive Care Medicine	England	Goal-directed therapy improves surgical outcomes by providing optimal fluid intake through cardiac monitoring.
Gutteridge G et al, [31]	2004	Anaesthesia & Intensive Care Medicine	England	Fluid administration expands the intravascular compartment, affecting effects depending on free transfer across the endothelium, contrasting crystalloid and colloid solutions.
Witt L et al, [33]	2012	Pediatric Anesthesia	Germany	Hemodilution with gelatin and hydroxyethyl starch (HES) significantly impaired clot formation compared to the isotonic crystalloid solution (ICS), requiring consideration when using high amounts.
Mauch J et al, [34]	2012	Pediatric Anesthesia	Switzerland	Fluids weaken clot strength, with HES and gelatine causing stronger impairments, while albumin and normal saline showed weaker fibrin polymerization.
Tommasino C et al, [36]	2007	Best Practice & Research Clinical Anaesthesiology	Italy	Haemodynamic stability and cerebral perfusion pressure are crucial for treating intracranial pathology. Fluid management requires understanding cerebral pathophysiology and considering its effects on the body.
Werner C et al, [37]	2007	British Journal of Anaesthesia	Germany	Secondary brain injury involves excitotoxic damage, inflammation, apoptosis, necrosis, and varying therapeutic options.
Mythen MG et al, [40]	2012	Perioperative Medicine	England	Increase mortality, hospital stay, admission, readmission, and patient-reported outcomes.

## Conclusions

Traumatic brain injury (TBI) is a greater community or social health problem that increases mortality and disability. Traumatic brain injuries can result from physical attacks, sports-related injuries, traffic accidents, etc. The development of therapeutic procedures for TBI recovery has been ongoing for many years, even though there is currently no effective treatment for TBI rehabilitation. For the best possible care, it is crucial to understand the various helpful therapy techniques in the pre-operative and pre-hospital phases. There are still questions concerning the benefits and dangers of perioperative fluid therapy. The rapid infusion of large crystalloids to restore blood volume and blood pressure is now the standard treatment for patients with combined traumatic brain injury (TBI) and hemorrhagic shock (HS). Crystalloid fluid therapy is more effective than colloid fluid therapy in patients with TBI. There are no standards of care or evidence-based guidelines for managing fluid treatment in patients having decompressive craniectomy.
